# Efficacy of erenumab and factors predicting response after 3 months in treatment resistant chronic migraine: a clinical service evaluation

**DOI:** 10.1186/s10194-022-01456-2

**Published:** 2022-07-22

**Authors:** Michael Lowe, Lesley Murray, Alok Tyagi, George Gorrie, Sarah Miller, Krishna Dani, the NHS Greater Glasgow and Clyde Headache Service

**Affiliations:** Institute of Neurological Sciences, Queen Elizabeth University Hospital, Glasgow, G51 4TF UK

**Keywords:** Migraine, Erenumab, Calcitonin gene-related peptide (CGRP), Monoclonal antibody

## Abstract

**Background:**

Calcitonin gene-related peptide (CGRP) inhibitors have been developed as options for treatment of chronic and episodic migraine. We present our experience of the use of erenumab in a tertiary headache centre.

**Methods:**

This was a prospective clinical audit of all patients commenced on erenumab following a locally agreed pathway and criteria over a consecutive period. Patients received monthly erenumab 140 mg for 3 months. Data were collected prospectively at baseline and 3 months follow up.

**Results:**

One hundred three patients were commenced on erenumab during the study period. Patients had tried a median of 7 previous prophylactics, including onabotulinum toxin A in 94%. At 3 months there was a reduction in median total (28 to 20, 29% reduction, *p* < 0.0001) and severe (15 to 5, 67% reduction, *p* < 0.0001) headache days. 39.8% of patients achieved at least a 30% reduction in total headache days; 61.8% of patients achieved at least a 50% reduction in severe headache days. Meeting either of these thresholds was considered a positive response, 68% of patients achieved this. Presence of daily headache pattern was negatively associated with response, (56% response vs. 90% without daily headache, *p* = 0.0003). There was no association between age, gender, presence of medication overuse or number of previously tried prophylactic treatments and response to erenumab. 43% of patients reported at least one adverse effect, most commonly constipation (26%); treatment was discontinued in 3 patients due to adverse effects.

**Conclusions:**

Erenumab was an effective treatment for chronic migraine in this treatment resistant population over 3 months of follow up. Presence of daily headache predicted poorer response but there was still a significant positive response rate in this group.

## Introduction

Chronic migraine is defined in the 3^rd^ edition International Classification of Headache Disorders (ICHD-3) as the presence of headache on 15 or more days per month for greater than 3 months, meeting the diagnostic criteria for migraine on at least 8 of those days [1]. This burden of headache on a nearly daily basis has a significant socioeconomic impact [2].

Frequent use of abortive therapies for migraine attacks, analgesics and triptans, can commonly lead to co-morbid medication overuse headache in patients with chronic migraine. Preventative therapies are therefore required for effective management.

A variety of classes of oral medication have an established role in migraine prevention. For patients who do not respond to these treatments, or are unable to tolerate them due to adverse effects, treatment options were previously limited, with botulinum toxin A being the mainstay of treatment in such patients. However, there remained a significant treatment gap for a proportion of patients who were refractory to all previously used evidence based migraine treatments.

Calcitonin gene-related peptide (CGRP) signalling is implicated in the pathophysiology of migraine attacks [3]. Recently monoclonal antibodies targeting either the CGRP ligand or receptor have been developed as treatments for migraine. In March 2019, the Scottish Medicines Consortium accepted erenumab for use on the National Health Service (NHS) Scotland in patients with chronic migraine who had not responded to/tolerated at least 3 prior preventative treatments [4].

We report our real-world experience of the use of erenumab in this previously treatment resistant population.

## Methods

We performed a clinical service evaluation of the use of erenumab in the first three months, to assess the effectiveness, tolerability and safety of erenumab in our routine clinical practice.

### Participants

In our headache service, based in the Department of Neurology, Queen Elizabeth University Hospital Glasgow (UK) we started using erenumab for the prevention of chronic migraine in March 2020. We considered patients for erenumab if the following treatments had been ineffective, not tolerated or contra-indictated: propranolol, amitriptyline, topiramate, candesartan, and botulinum toxin A. These patients were deemed to be treatment resistant. A condition of treatment was that medication overuse headache should have previously been addressed. We did not include patients with uncontrolled hypertension and those who were planning a pregnancy.

### Ethical approval

Under current NHS Health Research Authority guidance, service evaluation does not require research ethics committee review.

### Treatment protocol & data collection

Patients were referred by their treating clinician to a dedicated “CGRP clinic” run by either a headache clinical nurse specialist or a neuroscience pharmacist. At that appointment, baseline blood pressure was measured, suitability with respect to inclusion and exclusion criteria was reviewed, and baseline clinical and demographic data were recorded. This included previously tried preventative medications and current use of analgesics. Medication overuse was defined as ongoing use of paracetamol or non-steroidal anti-inflammatory drugs on 15 or more days per month and/or ongoing use of opiates or triptans on 10 or more days per month. Patients were taught injection technique at the same appointment.

Patients were required to have recorded a baseline headache diary, with continued daily recording whilst on treatment. As per the agreed pathway in the department, patients received erenumab at an initial dose of 140 mg every 4 weeks, delivered as a single subcutaneous injection using a pre-filled autoinjector. A 3 month supply of medication was delivered to patients’ homes using a homecare service. Patients were subsequently reviewed after 3 months of treatment in the nurse and pharmacist led “CGRP clinic”. Patient reported adverse effects were reviewed and a further blood pressure was recorded. Evidence of benefit was assessed using the completed headache diaries. A positive response was defined as at least a 30% reduction in mean monthly headache days, or a 50% or more reduction in mean monthly severe headache days. We defined a severe headache as being at least 7 out of 10 in severity, on the visual analogue scale. If patients had a positive response to erenumab, a further prescription for 9 months was issued. If patients had not responded, the medication was discontinued. Some patients with a borderline response or trend towards improvement were prescribed a further 3 month trial.

### Data analysis

An analysis of routine, prospectively acquired clinical data was performed. For purposes of diary analysis, a month was equivalent to a consecutive 4-week period (28 days). We analysed the data from the first three months or erenumab use for all patients starting the medication between March and December 2020, giving a total study population size of 103 patients. Statistical analysis was performed using GraphPad Prism [Version 9.3 for Windows, GraphPad Software LLC]. Pre- and post-treatment headache days and severe headache days data demonstrated significant skew deviating from the normal distribution, and therefore were compared Wilcoxon matched-pairs signed rank test. Blood pressure data were compared with paired t-test. Univariate analyses between responder and non-responder groups were performed: Fisher’s exact test was used to compare nominal variables; for non-parametric data the Mann–Whitney U-test was used to compare medians; where data were normally distributed the independent-samples t-test was used to compare means. Multiple logistic regression analysis was also performed including the same variables to assess for independent prediction of response at 3 months.

### Missing data

No patients were lost to follow-up over the 3 month period. However, a small number of patients had missing data at baseline or follow-up. These patients were excluded from analyses dependant on that data; the number of patients involved is reported individually per analysis. Patients who discontinued treatment early were not excluded and their headache diaries were analysed in the same manner as the rest of the cohort.

## Results

Baseline characteristics of the patient cohort are shown in Table 1. Patients had tried a median number of 7 previous preventative treatments, including botulinum toxin A in 94%.

**Table 1:** Baseline characteristics of the study population

Baseline Characteristics	Whole Cohort
Age (years, mean ± SEM)	44.4 ± 1.2
Gender (n [%] female)	88/103 [85%]
Number of previously tried preventatives (median [95% CI])	7 [7, 8]
Non-responder to botulinum toxin A (n [%])	97/103 [94%]
Current medication overuse	50/103 [49%]
Headache days (median [95% CI])	28 [28 – 28]
Severe headache days (median [95% CI])	15 [12-18]
Current daily headache pattern (n [%])	62/103 [60%]

*SEM* Standard error of the mean. *CI* Confidence interval

### Treatment completion

101 patients completed 3 doses of erenumab per the locally agreed protocol. 2 patients discontinued after 2 doses; one due to adverse effects and one due to worsening of headaches. These patients were not excluded and their completed 3 month headache diaries analysed in the same manner as the rest of the cohort.

### Monthly headache days

**Fig. 1:**
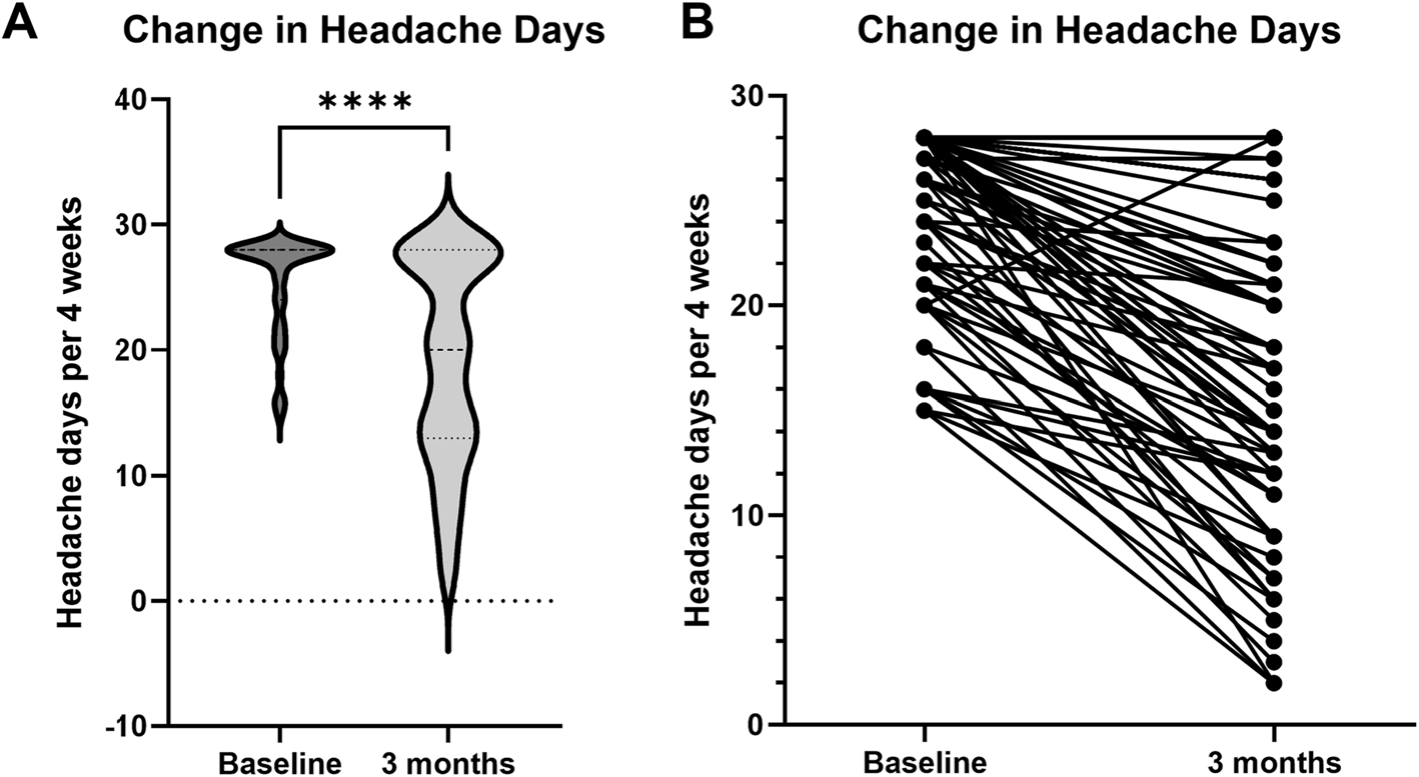
violin plot **(a)** and before-after individual values plot **(b)** showing headache days at 3 months compared to baseline. There was a significant reduction in headache days. In **(a)** dashed line shows median, dotted lines at upper and lower quartiles. ****p < 0.0001 by Wilcoxon matched-pairs signed rank test

Baseline and 3 month headache days data were available for all 103 patients. At 3 month follow up there was a significant reduction in headache days (Fig. 1), with median headache days of 28 (95% CI 28.0–28.0) at baseline compared with 20 (95% CI 16.0–22.0) at 3 month follow up (*p *< 0.0001 by Wilcoxon Signed-Ranks test).

### Monthly severe headache days

**Fig. 2:**
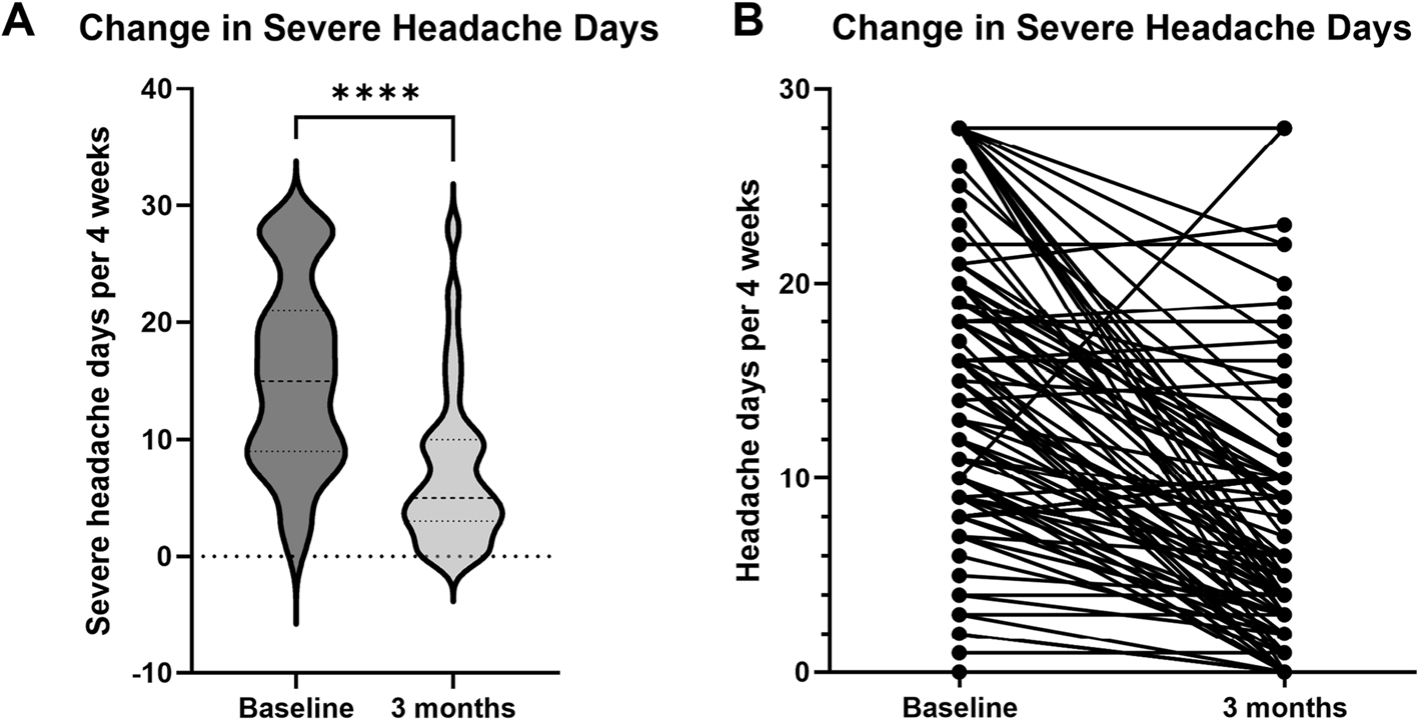
violin plot **(a)** and before-after individual values plot **(b)** showing severe headache days at 3 months compared to baseline. There was a significant reduction in severe headache days. In **(a)** dashed line shows median, dotted lines at upper and lower quartiles. ****p < 0.0001 by Wilcoxon matched-pairs signed rank test

Baseline and 3 month severe headache days data were available for 97 patients. At 3 month follow up there was a significant reduction in severe headache days (Fig. 2), with median severe headache days of 15 (95% CI 12.0–18.0) at baseline compared with 5 (95% CI 4.0–7.0) at 3 month follow up (*p* < 0.0001 by Wilcoxon Signed-Ranks test).

### Response rates

**Fig. 3:**
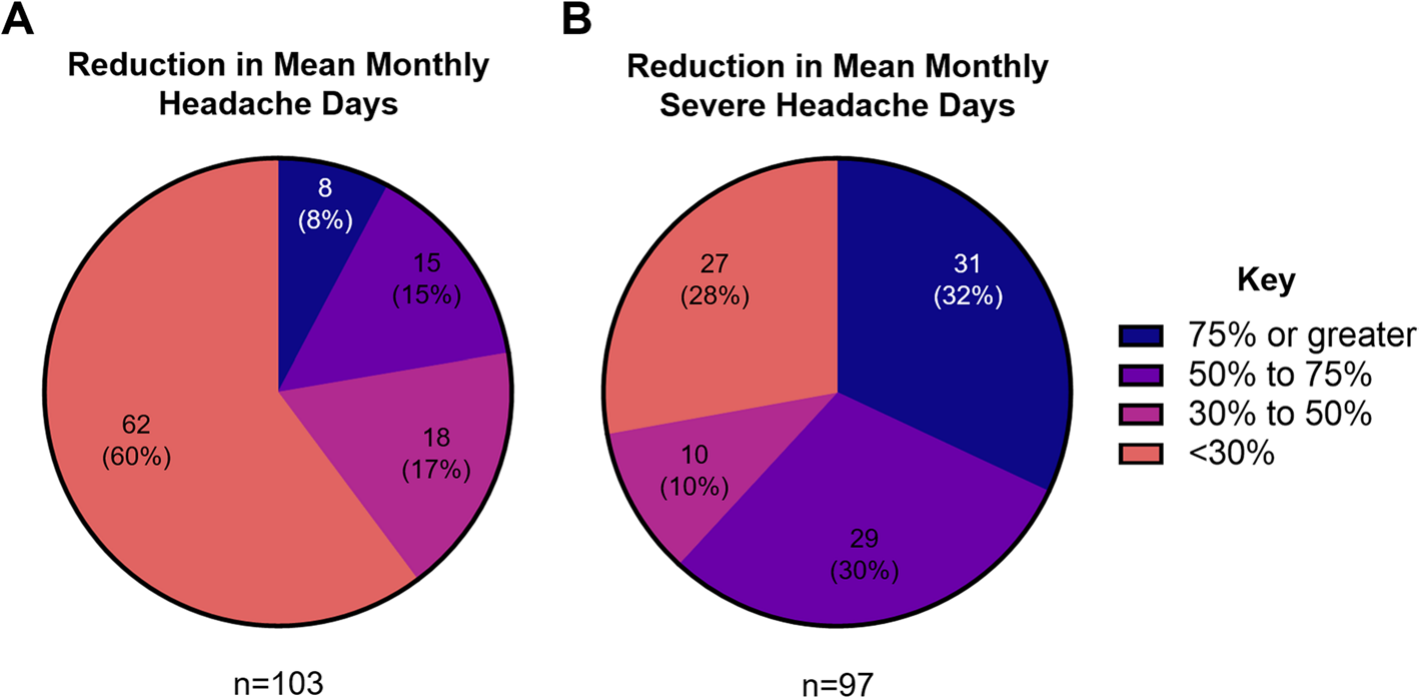
pie charts showing reductions in mean headache days (**a**) and severe headache days (**b**) observed over the study period compared to baseline

After 3 months of treatment with erenumab, at least 30%, 50% and 75% reductions in mean monthly headache days were achieved by 39.8%, 22.3% and 7.8% of patients respectively (Fig. 3(a)). At least 30%, 50% and 75% reductions in mean monthly severe headache days, in the 97 patients for whom baseline and follow up data were available, were achieved by 72.2%, 61.8% and 32.0% of patients respectively (Fig. 3 (b)).

Positive clinical response was defined as a 30% reduction in mean headache days or 50% reduction in mean severe headache days over the 3 month treatment period. In 5 patients insufficient baseline and/or follow up data were available to categorise their response and these patients were excluded.

**Fig. 4:**
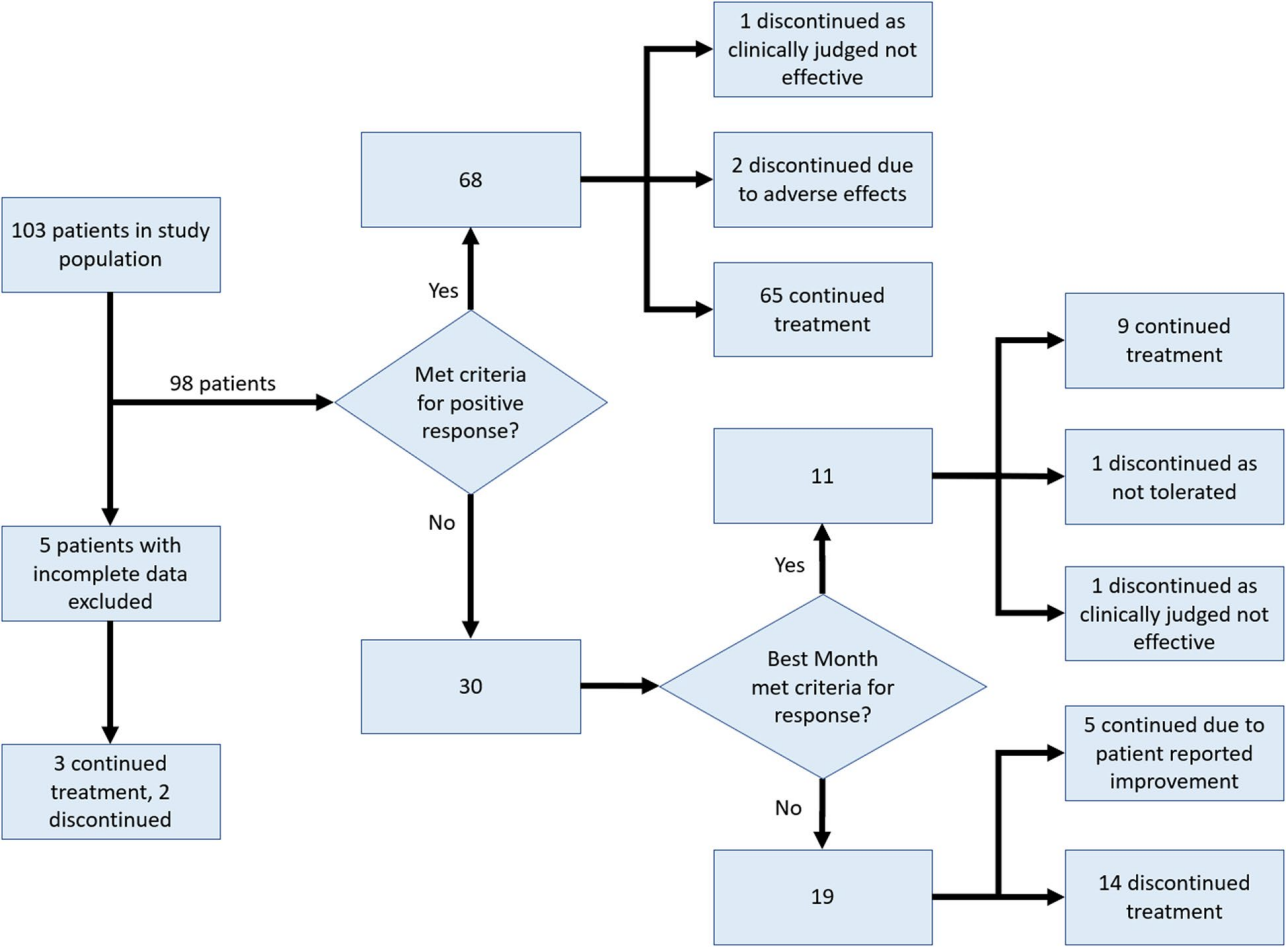
flow chart demonstrating overall positive response rate and subsequent clinical decision making. Positive response criteria were a 30% reduction in total headache days and/or a 50% reduction in severe headache days

Sixty-eight (69%) patients met the pre-defined criteria (30% reduction in headache days or 50% reduction in severe headache days) for a response to erenumab over the 3 month study period (Fig. 4).

### Treatment continuation

Some patients not meeting criteria for positive response using mean data over 3 months did meet these criteria in at least 1 month. For the purposes of clinical decision making regarding treatment continuation, this “best month” was used to assess response, to allow for patients showing a trend towards improvement. Using best month data, 79 patients met criteria for positive response. Of these 74 continued on treatment. 3 discontinued due to adverse effects. 2 discontinued as whilst meeting criteria the improvement in symptoms was not judged clinically significant. 19 patients did not meet criteria for a positive response. Of these 14 discontinued treatment. In 5 patients not meeting criteria for positive response there was nevertheless a patient reported significant improvement and treatment was continued for a further 3 month trial period.

### Predictors of response

Univariate analyses were performed to assess whether baseline variables of interest (age, gender, baseline headache days, number of previously tried treatments, medication overuse at baseline and daily headache pattern at baseline) were associated with reponse to erenumab (Table 2).

**Table 2:** Univariate analyses & multiple logistic regression comparing baseline characteristics between responders and non-responders

Univariate Analyses					Multiple logistic regression	
*Variable*	*Responders (n* = *68)*	*Non-responders (n* = *30)*	*Difference (95% CI)*	*p-value*	*Odds ratio* *(95% CI)*	*p-value*
Age (mean ± SEM)	44.8 ± 1.56	44.1 ± 2.16	-0.62 (-6.07 – 4.84)	*p* = 0.8230*	1.00 (0.96 to 1.04)	*p* = 0.8742
Gender (*n* [%] female)	57 [83.8%]	26 [86.7%]	OR 0.79 (0.26 – 2.66)	*p* > 0.9999†	1.07 (0.29 to 4.55)	*p* = 0.9240
Baseline headache days (median [IQR])	27 [22–28]	28 [28–28]	1 (0 – 2)	***p* = 0.0006**‡	1.03 (0.77 to 1.36)	*p* = 0.8058
Number of previously tried treatments (median [IQR])	7 [6.25–8]	7 [6-8]	0 (-1 – 0)	*p* = 0.3922‡	1.29 (0.90 to 1.87)	*p* = 0.1701
Any medication overuse at baseline (*n* [%])**	35/63 [55.6%]	13/28 [46.4%]	OR 1.44 (0.56 – 3.40)	*p* = 0.50†	1.41 (0.52 – 3.89)	*p* = 0.5048
Daily headache at baseline (*n* [%])	33 [48.5%]	26 [86.7%]	OR 0.15 (0.05 – 0.44)	***p* = 0.0003**†	0.11 (0.01 – 0.73)	***p* = 0.0395**

^*^ means compared with unpaired t-test

^†^ medians compared with Mann–Whitney U-test

^‡^ contingency assessed with Fisher’s exact test

*OR* Odds Ratio, *CI* Confidence interval, *IQR* Interquartile range

Non-responders had a greater number of headache days at baseline. Specifically, the presence of daily headache at baseline was found to be associated with a lower rate of positive response to erenumab. Rate of positive response in the daily headache group was 56% compared to 90% in the non-daily headache group. When patients with a daily headache pattern were excluded, the number of headache days was not significantly different between groups (*n* = 39, median headache days in responders 22 vs 22.5 in non-responders, *p* = 0.986); however this analysis is limited by the fact that only 4 patients remained in the non-responder group after excluding daily headache. No association was found between response and age, gender, number of previously tried treatments, previous failure of 8 or more treatments or presence of medication overuse at baseline.

A multiple logistic regression analysis was then performed including these variables. Only daily headache retained significance (OR 0.11 [0.01–0.72], *p* = 0.0395) in the final model when other variables were held constant.

### Adverse effects

44 patients (43%) reported at least one adverse effect, a breakdown of adverse effects is shown in Table 3. The commonest reported adverse effect was constipation in 27 patients. In two patients severe constipation resulted in discontinuation of treatment. A further patient discontinued treatment due to tremors and tachycardia. No other patient had to discontinue treatment due to adverse effects.

**Table 3:** Adverse effects reported during the 3-month treatment period. Some patients experienced multiple adverse effects. Other captures adverse effects reported by only a single patient, these included bloating, cold extremities, diarrhoea, joint pain, low mood, restless legs, weight gain, tremor and tachycardia

Adverse effect	Number reporting
Constipation	27 (26%)
Injection site reactions	4 (4%)
Dizziness/lightheadedness	3 (3%)
Hair loss	3 (3%)
Itch	3 (3%)
Rash	3 (3%)
Fatigue	2 (2%)
Muscle cramps	2 (2%)
Nausea	2 (2%)
Other	9 (9%)

### Blood pressure (BP)

Baseline and 3 month follow up BP recordings were available for 91 patients. There was a small but statistically significant increase in systolic BP of 2.4 mmHg at 3 months compared to baseline (to 123.5 ± 1.4 from 121.1 ± 1.3, p = 0.0255, mean ± SEM). There was no significant difference in diastolic BP (to 80.8 ± 0.9 from 81.7 ± 0.8, *p* = 0.1945).

## Discussion

This clinical service evaluation evaluates the efficacy, safety and tolerability of erenumab in a real-world clinical population. This population includes patients which present a familiar challenge to headache specialists. Patients had not responded to multiple oral prophylactics, and a significant majority had not responded to two cycles of botulinum toxin A. Medication overuse was reported in the majority of patients at baseline. The majority of patients also reported a chronic daily headache pattern.

### Response to erenumab

The results presented here show that erenumab is an effective and generally well-tolerated treatment option in this difficult-to-treat population. There is a more marked reduction in severe headache days than total headache days. Other observational studies in clinical practice which have reported both total headache days and migraine days have also reported greater reductions in migraine days than total headache days [5–8].

The proportion of patients achieving 30%, 50% and 75% reductions in mean monthly severe headache days (72.2%, 61.8% and 32.0% of patients respectively) is greater than that reported in the randomised clinical trial (RCT) in chronic migraine, with 50% and 75% response rates around 40% and 20% respectively being reported [9, 10].

Other studies of response to erenumab in ‘real-world’ clinical practice from the UK [5], US [6], the Netherlands [7], Italy [11, 12] and Australia [8] have reported 50% response rates (reduction in monthly migraine days) at 3 months ranging from approximately 30% to 58.8% [5–8, 11, 12]. Our results are above the upper end of this range. It is notable that in the study reported by Cheng et al., who found a 3 month response rate of 58.8%, 88% of patients received the 140 mg erenumab dose [8]. This is in contrast to the other reports with lower 3 month response rates, in which most patients were commenced on the 70 mg dose, often escalating to 140 mg at 3 months if they had not responded at this time point [5, 7, 11, 12]. It is possible that our high response rate may in part be attributed to all of our patients commencing on the 140 mg dose. Other factors including patient selection and differences in outcome measures (severe headache days being not necessarily equivalent to migraine days) could also account for our high response rate. Cheng et al. found that at 6 months follow up 50% response rate had decreased to 46.5% [8]; whilst Lambru et al. found an increase from 35 to 38% [5]. These opposite trends may again partially reflect the underlying dosing strategy.

In their review of RCT data, Ornello et al. suggested a slight advantage of 140 mg over 70 mg dosing, particularly in patients with prior preventative treatment failures (this applying to all patients in our cohort) [13]. Further work is needed to establish the best dosing strategy for erenumab and longer term follow up of our cohort will be helpful to establish whether any apparent benefit from an initial high dose is sustained.

### Predictors of response

Our evaluation for baseline characteristics that might predict response to erenumab revealed a striking novel negative association between the presence of a daily headache pattern and response to erenumab, with a 56% response rate in this group compared to 90% in the non-daily headache group. Prevalence of daily headache was high, at 60%, in our cohort. In the cohort reported by Cheng et al. the prevalence of daily headache (or ‘zero headache free days’) was 77/170 (45%), and in contrast to our study did not find any association between patients reporting no headache free days and response to erenumab at 3 or 6 months [8]. Whilst other studies have not specifically reported with respect to daily headache, they have generally shown that non-responders had greater numbers of headache days [11, 14], although Barbanti et al. found that a greater number of headache days was associated with positive erenumab response in their chronic migraine group [15]. This observation merits further investigation and we would encourage that this metric be included in future studies of response to erenumab. Whilst response rates were significantly lower in the daily headache group, it is important to note that 56% of this group did have a positive response. Therefore in clinical practice we would not preclude patients with daily headache from a trial of erenumab but instead temper expectations of response.

We did not find any association between age, gender, presence of medication overuse at baseline or number of previously tried prophylactic treatments and response to erenumab. The lack of impact of medication overuse on response is particularly encouraging as this is a frequent issue in this patient population and abortive treatment withdrawal can be difficult to achieve. Other studies have also found no association between medication overuse and response to erenumab [5, 8, 11, 16]. However, Baraldi et al., looking at duration of medication overuse rather than simply its presence or absence, found that greater duration of medication overuse was associated with a negative response to erenumab at 12 months [14].

Patients in our cohort had also generally not responded, or become non-responders after an initial response, to large numbers of preventative treatments. It is encouraging therefore that numbers of prior treatment failures were not associated with erenumab response, and this lack of association has been reported in other studies [8, 11]. In two studies with longer term follow up of 12 months, however, a negative association with response to erenumab has been reported with more prior treatment failures [14, 15]. One of these cohorts was reported earlier at 12 weeks of follow up and a significant association was not seen at this point [12], suggesting that an association with treatment-refractoriness may emerge over time. As further long-term follow up data becomes available this issue will hopefully become clearer but our early results are encouraging in this treatment resistant population.

### Tolerability of erenumab

The overall burden of adverse effects we found (43%) was similar to that reported in RCTs (ranging 44–57.3%) [10, 17–19]. In post-marketing studies constipation has emerged as the most commonly reported adverse effect with erenumab [5, 20, 21], and the a special warning is now included within the SmPC by the European Medicines Agency [22]. Our experience also supports this observation. Constipation reported with erenumab can be severe and in two patients in our study led to treatment cessation.

Due to the vasodilatory action of CGRP particular attention has been paid to the potential effects on blood pressure [23]. Clinical trials did not show a significant risk of hypertension with erenumab but it has been reported in post-marketing surveillance [24, 25] and is now included as a warning on the package insert in the US [26]. We found a statistically significant rise in systolic BP following 3 months of erenumab treatment but the magnitude of this was very small (2.4 mmHg) and therefore of questionable clinical significance. No patient discontinued treatment due to development of hypertension.

### Strengths and limitations

The main strength of this study is the real-world setting and the inclusion of a previously treatment resistant group of patients, 94% of which had not responded to botulinum toxin A, which is reflective of the challenges posed in chronic migraine management in clinical practice. The striking efficacy of erenumab in such a group of patients over a 3 month period is encouraging, but it will be important to assess whether this is sustained over a longer period of follow up in future. The main limitation is the uncontrolled, open label design which inherently introduces bias. Inclusion of all patients in the evaluation period reduces selection bias but does not eliminate this.

A more specific caveat is the use of monthly severe headache days, as opposed to the more commonly reported monthly migraine days (thereby capturing all days with head pain scored 7/10 or greater, rather than all days fulfilling ICHD-3 criteria for migraine). This was chosen as it is readily extracted from headache diaries and much simpler for patients to record. However, this metric does not capture other disabling migraine symptoms such as aura symptoms, nausea and motion, noise or light intolerance and therefore may over or underestimate the overall impact of treatment on quality of life. The difference in metric may also limit accurate comparison with other similar studies and it is possible this may account for the relatively high response rate seen here. Future studies could additionally utilise patient-reported outcome measures to better capture these important other dimensions of treatment response.

The sample size was not calculated due to the real-world nature of this evaluation, rather this was based on all patients with available baseline and follow-up data during the evaluation period. However, the more exploratory analyses of predictors of response resulted in some small subgroups, particularly given the small number of non-responders. Therefore these were likely underpowered to detect significant association and would require larger studies to further evaluate.

## Conclusions

We report a high response rate to erenumab at a 140 mg dose in a treatment resistant treatment population with a high prevalence of medication overuse and high headache burden. This response was independent of prior treatment failures and medication overuse. Presence of daily headache predicted poorer response but even in this population there was a good overall response. Erenumab was well tolerated with very few significant adverse effects leading to treatment cessation. Longer term follow up is needed to establish durability of this response, optimum dosing strategy and treatment duration.

## Data Availability

The anonymised datasets used and analysed during the current study are available from the corresponding author on reasonable request.

## Abbreviations

BP: Blood pressure
CGRP: Calcitonin gene-related peptide
ICHD: International Classification of Headache Disorders
NHS: National Health Service
RCT: Randomised controlled trial
SEM: Standard error of the mean
UK: United Kingdom
